# Membranoproliferative glomerulonephritis with predominant IgG2 and IgG3 deposition in a patient with IgG4-related disease

**DOI:** 10.1186/s12882-015-0164-8

**Published:** 2015-10-26

**Authors:** Kenji Ueki, Yuta Matsukuma, Kosuke Masutani, Akihiro Tsuchimoto, Kiichiro Fujisaki, Kumiko Torisu, Shigeru Tanaka, Tamotsu Kiyoshima, Satoshi Hisano, Takanari Kitazono, Kazuhiko Tsuruya

**Affiliations:** Department of Medicine and Clinical Science, Graduate School of Medical Sciences, Kyushu University, Fukuoka, Japan; Department of Oral Pathology, Graduate School of Dental Sciences, Kyushu University, Fukuoka, Japan; Department of Pathology, Faculty of Medicine, Fukuoka University, Fukuoka, Japan; Department of Integrated Therapy for Chronic Kidney Disease, Graduate School of Medical Sciences, Kyushu University, 3-1-1 Maidashi, Higashi-ku, Fukuoka, 812-8582 Japan

**Keywords:** Corticosteroid, Full-house immunofluorescence, IgG subclasses, Kidney biopsy, Salivary gland

## Abstract

**Background:**

IgG4-related disease is a novel disease entity characterized by elevated serum IgG4 and tissue infiltration by IgG4-positive plasma cells. Typical renal pathology is tubulointerstitial nephritis with storiform fibrosis, although the co-existence of various glomerular lesions has been described. Here, we present the first report of a case of IgG4-related kidney disease and membranoproliferative glomerulonephritis showing the discrepancy in IgG subclasses between the kidney interstitium and glomeruli.

**Case presentation:**

A 70-year-old Japanese woman was diagnosed with membranoproliferative glomerulonephritis and focal tubulointerstitial nephritis with IgG4-positive plasma cells. Immunofluorescence studies revealed predominant deposition of IgG3 and IgG2, but not IgG4 in the glomeruli. We administered oral prednisolone at 30 mg/day, and the abnormalities in urine and blood tests gradually resolved.

**Conclusion:**

In this case, different patterns of IgG subclasses detected in the glomeruli and interstitial plasma cells suggest overlapping immunologic abnormalities. The favorable clinical course in our patient suggests that steroid therapy is promising in cases of IgG4-related kidney disease accompanied by glomerulonephritis.

## Background

IgG4-related disease (IgG4-RD) is a novel disease entity characterized by elevated serum IgG4 and tissue infiltration by IgG4-positive plasma cells. Renal involvement is often observed in this disease, and the typical histopathological findings are tubulointerstitial nephritis with abundant IgG4-positive plasma cells and storiform fibrosis and sclerosis [1]. With regard to the glomerular lesions, membranous nephropathy is the most common type, and can develop with and without tubulointerstitial nephritis [2–4]. However, some other forms of glomerulonephritis, such as mesangial proliferative glomerulonephritis, IgA nephropathy, Henoch–Schönlein purpura nephritis (IgA vasculitis), endocapillary proliferative glomerulonephritis, and membranoproliferative glomerulonephritis (MPGN) have also been reported [3–8]. In the cases with membranous nephropathy, IgG4 deposition along the glomerular capillaries was demonstrated [8], suggesting that membranous nephropathy shares the same pathogenesis as systemic serological abnormalities and other types of organ involvement. However, the interpretation of the pathogenesis of other types of glomerulonephritis is still unclear.

Here, we report the case of a patient diagnosed with IgG4-RD, complicated with MPGN and accompanied by focal tubulointerstitial nephritis with IgG4-positive plasma cells. We also investigated IgG subclasses in the glomeruli, and found predominant deposition of IgG3 and IgG2, but not IgG4.

## Case presentation

A 70-year-old Japanese woman with a past history of bronchial asthma and paroxysmal atrial fibrillation was transferred to Kyushu University Hospital because of leg edema and insidious onset of proteinuria and microhematuria. She had presented with mild leg edema 8 months before, and was admitted to another hospital because of pneumonia. Then heavy proteinuria was identified. She had a history of polyarthralgia, although other symptoms such as skin rash, hair loss, oral ulcers, and Sicca syndrome were not evident.

On admission, the patient’s characteristics were: height 144 cm, body weight 38.7 kg, body temperature 36.9 °C, and blood pressure 120/46 mmHg. Physical examination revealed normal breath sounds, heart sounds, abdomen and nervous system, but signs of anemia in the palpebral conjunctiva and bilateral leg edema were found. Urinalysis revealed proteinuria (2+), and occult blood (2+). Urinary protein excretion was 2.3 g/day. Complete blood counts revealed: white blood cells 3050/μL, red blood cells 252 × 10^4^/μL, hemoglobin 8.2 g/dL, hematocrit 24.3 %, and platelets, 12.9 × 10^4^/μL, with no atypical leukocytes. Serum biochemical analyses revealed: total protein 6.9 g/dL, serum albumin 3.0 g/dL, blood urea nitrogen 33.0 mg/dL, serum creatinine 1.09 mg/dL (eGFR 43.2 mL/min/1.73 m^2^), sodium 141 mmol/L, potassium 5.1 mmol/L, chloride 115 mmol/L, calcium 8.3 mg/dL, phosphate 4.9 mg/dL, and C-reactive protein 0.21 mg/dL. Quantitative immunoglobulin analysis showed: IgG 2407 mg/dL (normal, 872–1815 mg/dL), IgA 114 mg/dL and IgM 53 mg/dL (59–269 mg/dL), and the additional measurement of IgG subclasses revealed IgG1 564 mg/dL (320–748 mg/dL), IgG2 1650 mg/dL (208–754 mg/dL), IgG3 214 mg/dL (6.6-88.3 mg/dL), and IgG4 1110 mg/dL (4.8–105 mg/dL). Serum levels of C3, C4 and total serum hemolytic activity (CH_50_) were 58 mg/dL (71–135 mg/dL), 9 mg/dL (11–34 mg/dL), and 32 U/mL (31.6–57.6 U/mL), respectively. Although rheumatoid factor was increased to 133 IU/mL (<15 IU/mL), antinuclear antibodies, anti-double-stranded DNA antibodies, anti-U1-ribonucleoprotein antibodies, myeloperoxidase anti-neutrophil cytoplasmic antibodies (ANCA), proteinase 3 ANCA, anti-glomerular basement membrane (GBM) antibodies, anti-Sjögren syndrome related antigen (SS)-A antibodies, anti-SS-B antibodies, circulating immune complexes, and cryoglobulins were all negative. Hepatitis B virus surface antigen, hepatitis C virus antibodies, and human T-lymphotropic virus 1 antibodies were negative. T-SPOT.TB assays for the diagnosis of latent tuberculosis was also negative. Abdominal ultrasonography and computed tomography scanning revealed the absence of systemic lymphadenopathy, and no abnormal findings in the pancreas, salivary glands, and thyroid gland, although ultrasonography showed mild enlargement of bilateral kidneys (right 10.3 × 4.4 cm, and left 10.8 × 4.1 cm).

**Fig. 1:**
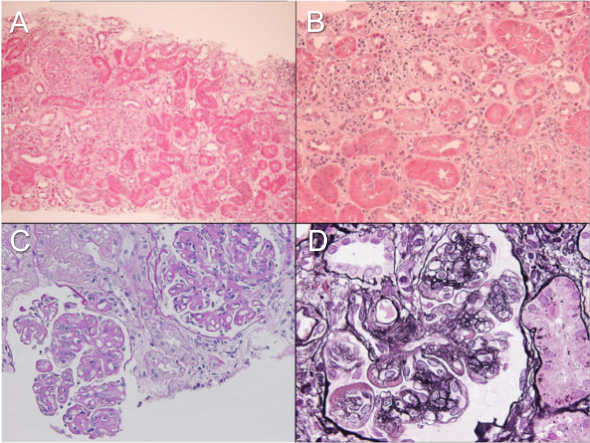
Light microscopic findings of kidney biopsy. **a** Kidney tissue showing focal interstitial inflammation and edema without evident storiform fibrosis. Hematoxylin and eosin (H&E) staining (magnification, × 100). **b** Interstitial infiltrating cells dominated by mononuclear cells, although infiltrating eosinophils and plasma cells are also observed. H&E staining (magnification, × 400). **c** Glomerulus showing mesangial proliferation, endocapillary hypercellularity, and subendothelial deposits. Periodic acid Schiff staining (magnification, × 400). **d** Double contours of glomerular capillaries are frequently observed. Periodic acid silver methenamine staining, (magnification, × 400)

**Fig. 2:**
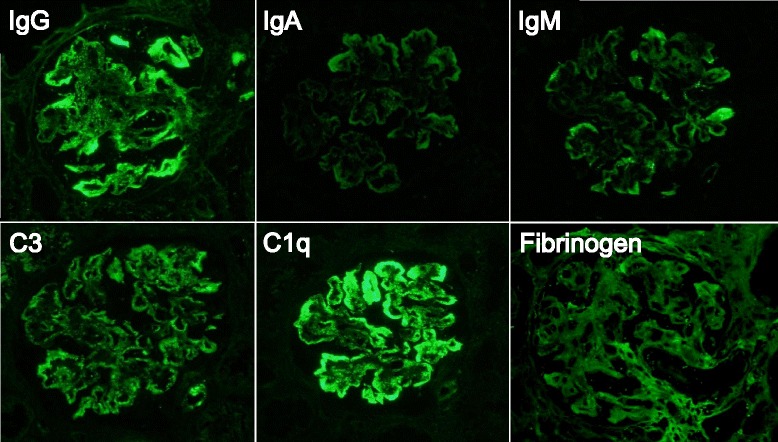
Immunofluorescence staining for immunoglobulin and complement. Glomerular depositions of IgG (3+), IgA (1+), IgM (1+), C3 (2+), C1q (3+) and fibrinogen (1+) are evident in the mesangial area and capillary loop

**Fig. 3:**
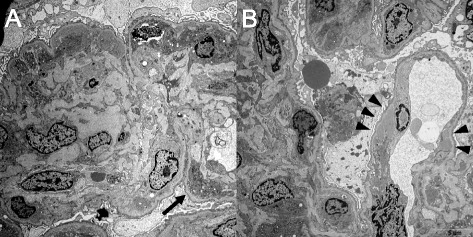
Electron microscopic findings. **a** Mesangial cell proliferation, mesangial matrix increase and mesangial electron dense deposit, and mesangial interposition (*arrow*) are noted. Subepithelial deposits were not found in the sampled glomeruli. **b** Diffuse foot process effacement and subendothelial electron dense deposit (*arrowhead*) are noted

Percutaneous kidney biopsy was performed on the 12th hospital day to determine the cause of the urinary abnormalities and kidney dysfunction. Light microscopic observation revealed global glomerulosclerosis in 1 of 13 glomeruli with mild interstitial edema and inflammation (Fig. 1a). The infiltrating cells were mainly mononuclear cells, but occasional eosinophils and a number of plasma cells were also noted. Storiform interstitial fibrosis was not evident (Fig. 1b). Twelve non-sclerotic glomeruli showed uniform changes; moderate to severe mesangial proliferation, increased mesangial matrix, and mild to moderate endocapillary hypercellularity. Subendothelial deposits (Fig. 1c), and double contours of the glomerular capillaries were also observed (Fig. 1d). No glomerular crescent was found. Immunofluorescence studies showed “full-house” staining for IgG (3+), IgA (1+), IgM (1+), C3 (2+), C1q (3+), and fibrinogen (1+) in the glomerular mesangium and the capillary wall (Fig. 2). Ultrastructural examination showed focal foot process effacement of the glomerular epithelial cells, mesangial and subendothelial electron-dense deposits, and mesangial interposition (Fig. 3). According to these findings, we confirmed the pathological diagnosis of MPGN type 1.

**Fig. 4:**
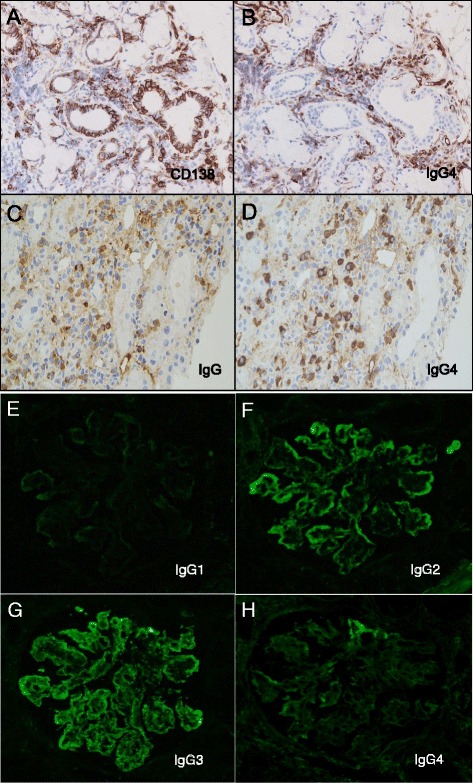
Immunohistochemical staining for IgG4 in lip and kidney biopsies. **a** Immunohistochemical staining for CD138 in a lip biopsy specimen. **b** IgG4 staining in the lip using serial section. In the glandular area, diffuse infiltration of IgG4-positive plasma cells is observed, and an IgG4/CD138 ratio is approximately 85 %. **c** Immunohistochemical staining for IgG in a kidney biopsy specimen. **d** IgG4 staining in the kidney using serial section revealed several foci of IgG4-positive cells in the interstitium. The IgG4/IgG ratio is approximately 90 %. Original magnification, × 400. **e**–**h** Glomeruli showing predominant IgG2 and IgG3 (IgG3 > IgG2) deposition in the mesangial area and capillary loop. Deposition of IgG1 is negative, and IgG4 shows weak and segmental deposition

Because the patient showed extremely high serum IgG4, we performed a lip biopsy and found the diffuse infiltration of IgG4 positive plasma cell in the glandular area (Fig. 4a and b); thus, the diagnosis of IgG4-RD was confirmed. Kidney-infiltrating plasma cells also produced IgG4, suggesting focal and early stage IgG4-related kidney disease (IgG4-RKD) (Fig. 4c and d). However, additional immunofluorescence studies revealed predominant deposition of IgG2 and IgG3, negative IgG1, and minimal IgG4 deposition in the glomeruli (Fig. 4e–h).

**Fig. 5:**
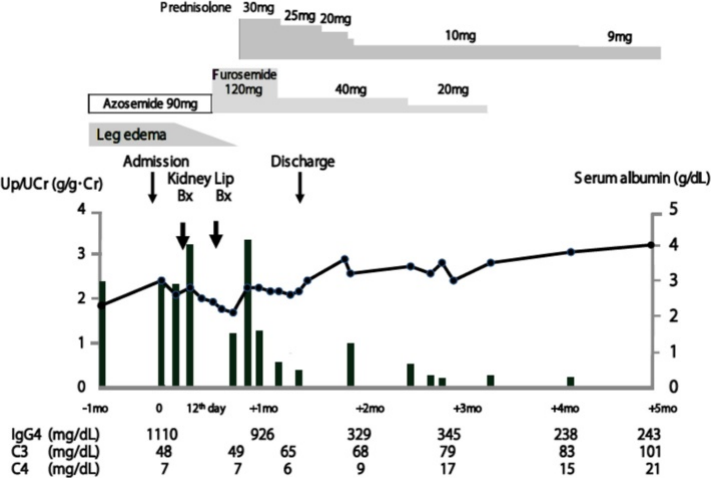
Clinical course of this patient. Up/UCr; urinary protein/creatinine ratio, Bx; biopsy

Although we found discrepancies in IgG subclass among the glomeruli and kidney interstitium, we treated this patient as IgG4-RD. Oral prednisolone administration (initial dose 30 mg/day) was started. The patient showed decreased urinary protein, and increased serum albumin and complement levels. In examinations conducted in our outpatient clinic 8 months later, the patient showed proteinuria (±), hematuria (3+), white blood cell count 9690/μL, hemoglobin 11.4 g/dL, platelet count 32.5 × 10^4^/μL, serum albumin 3.9 g/dL, serum creatinine 0.96 mg/dL, C3 99 mg/dL, C4 23 mg/dL, IgG 1002 mg/dL and IgG4 200 mg/dL (Fig. 5).

## Discussion

Here, we report the case of a patient diagnosed as IgG4-RD accompanied by MPGN type 1 with “full-house immunofluorescence”. We confirmed the diagnosis of IgG4-RD with serological tests and a lip biopsy showing significant infiltration of the glandular area by IgG4-positive plasma cells. IgG4-positive plasma cells with little fibrosis were also found in the kidney interstitium, and the IgG4/IgG ratio exceeded 40 %. We then diagnosed IgG4-RKD equivalent to stage A tubulointerstitial nephritis according to the schema suggested by Yamaguchi et al. [9]. However, the predominant IgG2/IgG3 deposition and minimal IgG4 deposition in the glomeruli conflicted with the systemic serological status, and the pathology of minor salivary glands and kidney interstitium. In this case, the most important differential diagnosis was a proliferative form of lupus nephritis because the patient developed hypocomplementemia and pancytopenia. However, her extrarenal symptoms did not suggest systemic lupus erythematosus (SLE), and laboratory data revealed that she was negative for antinuclear antibodies and anti-DNA antibodies; thus, ultimately, the patient did not fulfill the criteria of SLE.

Renal involvement in IgG4-RD, designated IgG4-RKD, is characterized by increased IgG4-positive plasma cells in the kidney interstitium, and various degrees of storiform fibrosis that can be recognized with methenamine silver and trichrome stains. Several forms of concurrent glomerulonephritis have been reported in IgG4-RKD [3–8]. Membranous nephropathy is the most common, and predominant IgG4 deposition along the glomerular capillary has been demonstrated [8], suggesting a common pathophysiology with systemic IgG4 overexpression and related cytokine production. However, in patients with IgG4-RD with other forms of glomerulonephritis, the common pathophysiology between systemic abnormalities and glomerular lesions is less apparent. One case of IgG4-RD and MPGN has been reported previously, but unfortunately, immunofluorescence analysis of IgG subclasses in the glomeruli was not included [5]. Thus, this is the first report of a case of IgG4-RKD and MPGN showing the discrepancy in IgG subclasses between the kidney interstitium and glomeruli.

The immunological abnormalities associated with IgG4-RD have been extensively investigated, and recent studies suggest an important role of T-helper (Th) 2 cells and regulatory T cells (Treg) in the development of IgG4-RD. Müller et al. demonstrated increased interleukin (IL)-4 and IL-5 levels, and upregulated IL-13 mRNA in bile samples from a patient with IgG4-related cholangitis [10]. Tanaka et al. investigated the expression patterns of cytokines, chemokines, and cytokine receptors in the labial salivary glands of patients with Mikulicz disease and Sjögren syndrome, and demonstrated that mRNA levels of IL-4, IL-5, IL-10, TGF-β, and Foxp3 were upregulated in Mikulicz disease compared with Sjögren syndrome and controls [11]. In the present case, predominant deposition of IgG2 and IgG3, but not IgG4 was found in the glomeruli, and immunofluorescence analysis showed that IgG3 deposition predominated. IgG3 is well-known as the typical “Th1 subclass IgG” and associated with primary MPGN [12, 13]. In general, Th1 and Th2 cells suppress each other, and polarization toward a Th1 nephritogenic immune response is associated with proliferative, crescentic forms of glomerulonephritis such as MPGN, ANCA-associated glomerulonephritis, and anti-GBM antibody glomerulonephritis, whereas Th2 activation is found in membranous nephropathy [12, 14, 15]. The renal pathology of the present case revealed co-existence of a Th1-associated pro-inflammatory response in the glomeruli, and Th2/Treg-associated changes in the interstitium. Similar phenomena of overlapping different pathologies can be found in the exceptional cases of crescentic membranous nephropathy with or without ANCA [16, 17]. Furthermore, in lupus nephritis, co-incidence of class IV and class V glomerular lesions are often found [18]. Further investigations are required to clarify the detailed mechanisms of the co-existence of two different immune responses in the same patient.

With regard to the treatment, we initially started low-dose oral prednisolone (30 mg/day) which was previously reported for the treatment of IgG4-RKD [3, 8], after we had carefully excluded SLE, cryoglobulinemic vasculitis, and monoclonal immunoglobulin deposition disease. Although low-dose steroid monotherapy is not sufficient for primary MPGN type 1, the patient’s urinary protein immediately decreased, kidney function was preserved, and serum IgG4 levels showed a gradual decline. The complement levels and complete blood cell counts also returned to the normal range. In the previous report of MPGN and IgG4-RKD reported by Morimoto et al. [5], the patient died of mycoplasma pneumonia and congestive heart failure a few months after the biopsy confirmation and without receiving corticosteroids. Thus, the favorable clinical course in our case is promising, and it is important to collect information on more cases of IgG4-RKD accompanied by glomerulonephritis to determine the response of such patients to steroid therapy.

## Conclusions

We have described the case of a patient with IgG4-RD accompanied by IgG4-related tubulointerstitial nephritis and MPGN with predominant IgG2 and IgG3 deposition. Different patterns of IgG subclasses detected in the glomeruli and interstitial plasma cells suggest overlapping immunologic abnormalities. IgG4-RD is a novel disease entity and the detailed pathophysiology is still unknown; thus, this emphasizes the need to accumulate the details of more cases of IgG4-RKD, and perform IgG subclass staining extensively if glomerular lesions are found in IgG4-RKDs to clarify the nature of this disease.

## Consent

Written informed consent was obtained from the patient for publication of this Case report and any accompanying images. A copy of the written consent is available for review by the Editor of this journal.

## Abbreviations

ANCA: Anti-neutrophil cytoplasmic antibodies
IgG4-RD: IgG4-related disease
IgG4-RKD: IgG4-related kidney disease
IL: Interleukin
MPGN: Membranoproliferative glomerulonephritis
SLE: Systemic lupus erythematosus
SS: Anti-Sjögren syndrome related antigen
Th: T-helper
Treg: Regulatory T cells
